# A greener Greenland? Climatic potential and long-term constraints on future expansions of trees and shrubs

**DOI:** 10.1098/rstb.2012.0479

**Published:** 2013-08-19

**Authors:** Signe Normand, Christophe Randin, Ralf Ohlemüller, Christian Bay, Toke T. Høye, Erik D. Kjær, Christian Körner, Heike Lischke, Luigi Maiorano, Jens Paulsen, Peter B. Pearman, Achilleas Psomas, Urs A. Treier, Niklaus E. Zimmermann, Jens-Christian Svenning

**Affiliations:** 1 Landscape Dynamics, Swiss Federal Research Institute WSL, Birmensdorf, Switzerland; 2 Plant Ecology Unit, Botany, Department of Environmental Sciences, University of Basel, 4056 Basel, Switzerland; 3 Department of Geography, University of Otago, Dunedin, New Zealand; 4 Department of Bioscience, Aarhus University, Roskilde, Denmark; 5 Arctic Research Centre, Aarhus University, Aarhus, Denmark; 6 Ecoinformatics and Biodiversity Group, Department of Bioscience, Aarhus University, Aarhus, Denmark; 7 Department of Bioscience, Aarhus University, Kalø, Rønde, Denmark; 8 Department of Geosciences and Natural Resource Management, University of Copenhagen, Denmark; 9 Department of Biology and Biotechnologies ‘Charles Darwin’, University of Rome ‘La Sapienza’, Italy

**Keywords:** Arctic, climatic niche modelling, climate change impact, disequilibrium, postglacial re-colonization, shrub expansion

## Abstract

Warming-induced expansion of trees and shrubs into tundra vegetation will strongly impact Arctic ecosystems. Today, a small subset of the boreal woody flora found during certain Plio-Pleistocene warm periods inhabits Greenland. Whether the twenty-first century warming will induce a re-colonization of a rich woody flora depends on the roles of climate and migration limitations in shaping species ranges. Using potential treeline and climatic niche modelling, we project shifts in areas climatically suitable for tree growth and 56 Greenlandic, North American and European tree and shrub species from the Last Glacial Maximum through the present and into the future. In combination with observed tree plantings, our modelling highlights that a majority of the non-native species find climatically suitable conditions in certain parts of Greenland today, even in areas harbouring no native trees. Analyses of analogous climates indicate that these conditions are widespread outside Greenland, thus increasing the likelihood of woody invasions. Nonetheless, we find a substantial migration lag for Greenland's current and future woody flora. In conclusion, the projected climatic scope for future expansions is strongly limited by dispersal, soil development and other disequilibrium dynamics, with plantings and unintentional seed dispersal by humans having potentially large impacts on spread rates.

## 1. Introduction

Arctic vegetation is changing in response to increasing temperatures over the past decades [1]. Satellite imagery indicates increased productivity [2], while repeated-photographic studies report greater shrub cover [3,4], increased tree growth at the boreal–tundra ecotone [5], and northward expansions of trees [6,7]. These vegetation changes will trigger several feedback loops with the climate system [8] and may have profound effects on ecosystems [9]. Do these changes mark the beginning of a greener future Arctic in which tundra vegetation is transformed by the expansion of a rich boreal woody flora, similar to the situation during Pliocene and certain Pleistocene warm periods (cf. [10,11])? Recent evidence suggests that shrubs are currently expanding locally across the entire Arctic, although at regionally varying rates [12]. Possible mechanisms explaining such regional variation include soil disturbance and changes in biotic interactions such as herbivory, while dispersal limitation has received very limited attention. Nonetheless, dispersal dynamics could be important and especially so in certain parts of the Arctic, such as Greenland owing to its isolated position, rugged topography [13,14] and massive inland icecap.

Greenland's current vegetation is dominated by Arctic tundra, with subarctic forest–tundra vegetation only occurring inland in southern Greenland [15,16]. The flora of Greenland has relatively few vascular plant species relative to other Arctic regions [17], with currently only four native tree and large shrub species, *Sorbus groenlandica, Alnus viridis* ssp. *crispa, Betula pubescens* and *Salix glauca*, and the low-growing *Juniperus communis* being the only conifer presently native to Greenland. This floristic poverty may not only reflect the contemporary climatic conditions, but also persistent historical effects of Greenland's nearly full ice cover during recent glaciations, largely prohibiting *in situ* survival, and its isolated position, limiting postglacial immigration.

**Figure 1.**
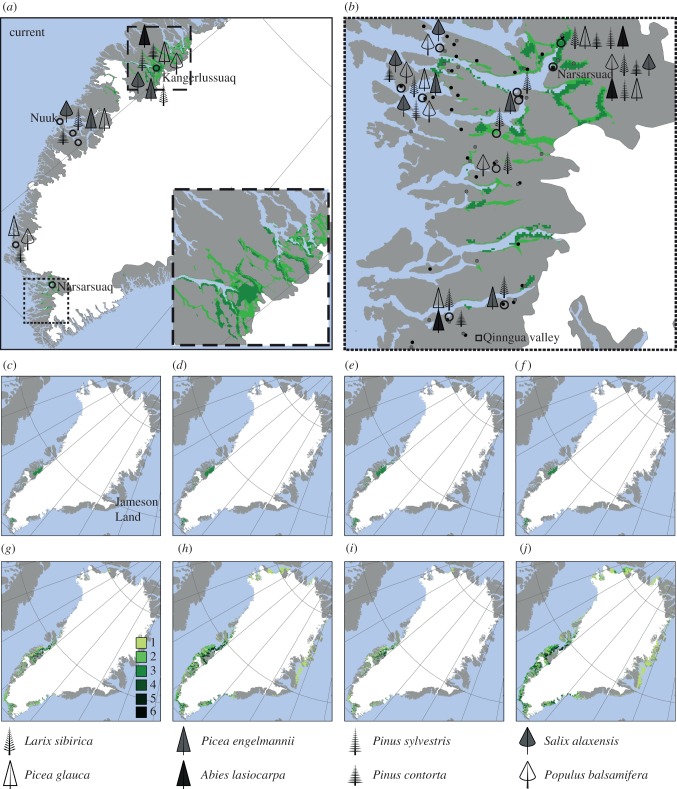
Areas suitable for trees according to the treeline model as well as current natural and planted occurrences of selected native and non-native tree and shrub species. (*a*,*b*) Current projections of the treeline model with two Digital Elevation Models of different resolution: 30-m (light green) and 30″ (approx. 700-m, dark green). Black circles indicate areas where non-native trees or shrubs have been planted (see the electronic supplementary material, appendix S2 for details). Qinngua valley: locality with natural woodland vegetation mentioned in the text. In (*b*), black and grey dots represent known native occurrences of *B. pubescens* and *So. groenlandica,* respectively. (*c*–*f*) Projections of suitable areas for tree growth at (*c*) 2 kyr ago, (*d*) 4 kyr ago, (*e*) 6 kyr ago and (*f*) 9 kyr ago, respectively. (*g*–*j*) Future projections: (*g*) A1b, 2050, (*h*) A1b, 2100, (*i*) A2, 2050 (*j*) A2, 2100. Past and future projections at the 30″ resolution. The number of GCMs for which suitable conditions are projected is shown.

A number of introduced North American and Eurasian tree and shrub species are currently growing and reproducing in Greenland [18,19] (figure 1), suggesting that Greenland's woody flora may be in disequilibrium with climate by lacking some species that could physiologically occur there due to dispersal constraints on their establishment in Greenland. Dispersal limitation is an increasingly recognized cause of disequilibrium dynamics [20] (i.e. directional climate-driven vegetation changes that occur with a lag relative to the climatic driver [21]), and it is obvious that long generation times, barriers, habitat fragmentation, soil development and competition might slow down migration rates tremendously [22]. Notably, dispersal limitation and related disequilibrium dynamics are reported for the range limits of some northern tree species at the boreal-Arctic treeline [14,23,24] as well as within the boreal zone [25]. However, the extent to which the current Greenlandic tundra vegetation is the result of postglacial dispersal constraints on the migration and subsequent spread of shrubs and trees within the region remains unknown. A better understanding of these warming-induced vegetation dynamics is crucial for improving our ability to project vegetation changes and resulting feedbacks in Greenland under a warmer future climate.

The vegetation history of Greenland provides evidence for pronounced vegetation changes in response to increased temperatures. In the Late Pliocene and beginning of the Pleistocene (*ca* 2 Myr ago), Greenland was at times almost ice-free and boreal forests expanded across large areas [10]. Macrofossil remains from northeastern Greenland show the presence of subarctic forest–tundra with a rich boreal shrub and tree flora, consisting of species and genera such as *Picea mariana, Thuja occidentalis, Taxus, Betula* sect. *Albae, A. viridis* ssp*. crispa* and the now extinct *Larix groenlandii* and *Myrica arctogale* [11,26,27]. In some Middle Pleistocene interglacial episodes, southern Greenland was also covered by rich boreal forest, including species of *Picea, Abies*, *Pinus*, Taxaceae, *Alnus* and probably also *Betula* and *Populus* [28,29]. During the Last Interglacial (*ca* 130–100 kyr ago) when summer temperatures in Greenland were 4–6°C warmer than at present [10], *Alnus* cf. *viridis* ssp. *crispa* and *B. pubescens* [30] reached as far north as Jameson Land in central eastern Greenland [30], while vegetation in southern Greenland was dominated by *Alnus* and the temperate fern *Osmunda*. However, boreal conifers such as *Picea* were not present or at least not common [28]. Only a small subset of the above-mentioned species occurs in Greenland today. Cold temperatures and the large icecap of the last glaciation allowed just a few Arctic species to persist [31,32]. By the beginning of the Holocene, most of the coastline became ice free and by 6 kyr ago, sea level and ice volumes were close to present-day conditions [10], with higher summer temperatures than at present [2,10]. The large majority of the present flora of Greenland colonized postglacially from adjacent areas in North America and Eurasia [33]. Some woody species such as *Empetrum nigrum* s.l. and *Vaccinium uliginosum* recolonized Greenland from the start of the Holocene, 10–11 kyr ago, while long immigration lags are suggested by the later arrival of others, *Salix arctica, Sa. glauca, Betula nana* and *J. communis ca* 8–9 kyr ago, *Betula glandulosa ca* 6 kyr ago, and *A. viridis* ssp. *crispa* and *B. pubescens ca* 4 kyr ago [34,35].

Here, we assess the postglacial and likely future geographical responses of woody vegetation and species to climatic warming in Greenland by a combination of physiology-based treeline modelling, climatic niche modelling, migration modelling, and analyses of potential source and sink areas for immigrant woody species. First, we address the importance of climate and postglacial migration lags as constraints on the current distributions of trees and shrubs in Greenland. We do this by estimating (i) areas below the potential treeline, (ii) the degree to which Greenlandic species occupy climatically suitable areas within Greenland, (iii) which North American and European species could potentially grow in Greenland today, and (iv) when during postglacial times climate became suitable for these species in Greenland, allowing us to estimate immigration lag times. Second, we consider future climate scenarios to quantify the climate potential for twenty-first century expansions of tree and shrub species across Greenland and assess the likelihood that Greenland will be transformed again by a re-colonization of a rich boreal woody flora, similar to that occurring during the Pliocene and some Pleistocene warm periods. We do this by (i) forecasting treeline shifts and future climatically suitable areas for tree and shrub species, (ii) computing future migration lags and areas likely colonized by year 2100, based on specific dispersal distances and migration rates, and (iii) estimating extent of and distance to areas with analogous climates in Greenland, North America and Europe to highlight areas most likely to become sources or sinks for future immigrant or introduced woody species.

## 2. Material and methods

### (a) Study species

We focused our analyses on 12 shrubs and trees native to Greenland, as well as woody species with maximum heights greater than or equal to 50 cm which occur in Arctic or subarctic areas bordering Greenland, i.e. in North America and Europe (*n* = 26; see the electronic supplementary material, appendix S1 for further information). Furthermore, we selectively added some of the non-native tree and shrub species that are planted in Greenland today (*n* = 16), as well as a few taxa that occurred in Greenland during the Late Pliocene and in warm interglacials during the Pleistocene (*n* = 2). In total, 56 species were analysed.

We used three types of information on the study species' current geographical ranges: occurrence records extracted from GBIF (http://www.gbif.org), dot maps and range outlines (see the electronic supplementary material, appendix S1 for sources of the latter two). GBIF occurrences were filtered by selecting records with geographical positions from either direct observations or specimen records, with a horizontal precision of the geographical coordinates smaller than the resolution of the climatic information (less than or equal to 8 km). The precision of the geographical coordinates (as defined in [36]) was estimated by taking into account the number of decimal digits of the latitude and longitude and the position on the Earth with the Harvesine formula. To reduce the effects of bias in GBIF occurrences, we randomly spaced the samples by only including at most three records per 100 × 100 km. After digitalizing dot maps and range outlines, we sampled within each range outline presences by a geographically stratified random sampling (allowing one record per 100 × 100 km). Pseudo-absences were sampled outside the range outlines but only within the continents where the species naturally occur by random sampling of one absence per 100 × 100 km (see the electronic supplementary material, appendix S3). In addition to the information on native ranges, we compiled information on planting locations and establishment success of tree and shrub species in Greenland (see the electronic supplementary material, appendix S2).

### (b) Climatic data

Data on present-day climate were obtained from the Worldclim dataset at 5′ and 10′ resolution (period 1950–2000, http://www.worldclim.org/). Simulations of past climate were obtained from a global ocean–atmosphere climate model with a temporal resolution of 1000 years and a spatial resolution of 3.75° × 2.5° [37]. We selected several time points to cover the late glacial and Holocene, starting with the Last Glacial Maximum (*ca* 21 kyr ago) and including 15, 12, 9, 6, 4, 2 kyr ago. The simulations of past climate were first downscaled to 10′ resolution as described in [38] and subsequently disaggregated to 5′ resolution. Climate data for the future were obtained from the IPCC Data Distribution Centre (http://www.ipcc-data.org/) and the WCRP Multi-Model Data (https://esg.llnl.gov:8443/data) and initially downscaled to 30″ with the Worldclim (http://www.worldclim.org) climate grids as baseline and subsequently aggregated to 5′ resolution. Six global circulation models (CGCM v. 3.1, CSIRO-MK v. 3.5, GFDL-CM v. 2.1, ECHAM5/MPI, PCM, UKMO-hadCM3) and two emission scenarios (A1B and A2) [39] represented potential future climate. All six GCMs were used to analyse future treeline shifts, while only the CGCM 3.1 was used in the climatic niche models. We mostly considered two future time periods: 2041–2050 and 2091–2100, hereafter, referred to as 2050 and 2100, but used decadal time steps for simulating migration with MigClim [40]. Based on monthly values of temperature and precipitation we derived three bioclimatic variables that are regarded as important in determining subarctic and Arctic plant species distributions: average summer temperature, temperature of the coldest month and annual sum of precipitation. All climate rasters were projected to the North Pole Lambert Azimuthal Equal Area projection.

### (c) Estimating the potential climatic treeline

The potential climatic treeline was estimated by an algorithm that combined two criteria for the establishment of tree populations: (i) a minimum of 94 days with a daily mean air temperature of 0.9°C) and (ii) a mean air temperature during these days of at least 6.4°C [41,42]. First, daily mean temperatures at elevations ranging from 0 to 2000 m with a 25 m interval were computed based on monthly average temperatures (10′ resolution) and a standard adiabatic lapse rate of 0.55 K per 100 m. Second, the highest elevation meeting all the above-mentioned criteria was kept as the potential treeline for each of the 10′ grid cells of Greenland. Third, areas below the potential treeline were identified based on a Digital Elevation Model (DEM). The approach described above is hereafter referred to as the treeline model. We used two DEMs of different resolution. The 30″ SRTM DEM (obtained from http://www.worldclim.org) was used for all areas and times. With such coarse resolution, the treeline model might fail to identify small areas with suitable microtopographic conditions in regions with large elevation differences. Therefore, we applied the 30 m ASTER Global Digital Elevation Model (obtained from http://asterweb.jpl.nasa.gov/) in certain smaller regions under current conditions. These latter, computationally intensive, analyses were not possible for all of Greenland but, as expected, showed less restrictive areas of tree growth (figure 1) and suggest that the 30″ (approx. 700-m) projections through time probably provide conservative estimates. While the treeline definition used here follows [42], it is to some degree a matter of convention and individual trees might find suitable climate or microclimate outside the areas identified by the model.

### (d) Climatic niche modelling

Climatic niche models were calibrated with the 5′ resolution data and five commonly used algorithms, two machine learning (random forest and Maxent), one parametric (generalized linear models) and one semi-parametric (generalized additive models) logistic regression, and one simple rectilinear envelope approach (bioclim) [43]. Presences and pseudo-absences were weighted to contribute equally to final models [44]. Model evaluation was carried out by splitting the data in 80 per cent for calibration and 20 per cent for validation, and by using the true skill statistic (TSS) [45], the area under the receiver-operating characteristic curve (AUC) [46] and sensitivity. Model performance was generally good (AUC, 0.88 ± 0.05; TSS, 0.68 ± 0.12; sensitivity, 89.9 ± 3.9) and models calibrated on the full occurrence dataset (see electronic supplementary material, appendix S3) were used to project past, current and future suitable climatic areas. Current projections covered the Northern Hemisphere north of 30° latitude while all projections of past and future suitable climate were computed only within Greenland. We transformed the projected probabilities into presence–absence using the TSS for optimizing thresholds for splitting. We built simple summed ensembles of the projections and generally considered the agreement among the majority of the models (at least three) to provide high support for presence. If not noted otherwise, the reported results are based on the majority of models. All models were computed with biomod2 [47] in R v. 2.15.1 [48], and for Maxent, we used v. 3.3.3 k [49].

### (e) Likely colonized area, migration lags and required migration rates

To shed light on the likelihood of tree and shrub expansion in Greenland in the near future, we used three approaches: we (i) estimated past immigration lags based on pollen-based arrival dates [34,35] and when during postglacial times climate became suitable for native and non-native species in Greenland, (ii) simulated the likely spread and likely colonizable area of native species by year 2100 with estimated dispersal distances and a cellular automaton, MigClim [40], and (iii) used observed migration rates and a simple distance-based approach to compute future likely colonized areas (suitable areas that can be reached by year 2100 given a realistic migration rate), migration lags (how long it will take suitable areas to be reached by year 2100 given a realistic migration rate), as well as migration rates required to reach a given area by year 2100. See electronic supplementary material, appendix S5 for details on these analyses.

### (f) Areas of analogous climates

We identified and mapped areas in North America and Eurasia with analogous climates in Greenland, as well as areas in Greenland with analogous climates on the two continents. Following the methodology used in Ohlemüller *et al.* [50], we used a range of climatic niche breadths to quantify the extent of and distance to climatically analogous areas; narrow to wide niche breadths indicate climatically analogous areas for species with a narrow to wide climatic tolerance range. We restricted searching for analogous climates to the mainly boreal and Arctic climate zones north of 30°N (see the electronic supplementary material, appendix S4). Using the same climate variables as for the niche models, each grid cell in North America and Eurasia was compared with each grid cell in Greenland and each grid cell in Greenland was compared with each grid cell in North America and Eurasia (see the electronic supplementary material, appendix S4 for details).

## 3. Results

### (a) Potential treeline in Greenland

Current areas below the potential treeline at the 30″ and 30 m resolution mainly occur in southern Greenland and around Kangerlussuaq, inland in western Greenland (figure 1). These areas generally overlap or are close to the known localities of natural woodland and forest plantations, but are considerably larger in some regions and fail to project certain plantation and woodland sites in others, e.g. the ‘Kussuaq plantation’ and the natural woodland vegetation in Qinngua valley in southwest Greenland. Potential areas for tree growth are estimated to first appear at 9 kyr ago and are expected to expand considerably towards year 2100 (figure 1), with three of six GCMs even projecting suitable areas for tree growth in northern Greenland.

### (b) Climatically suitable areas and migration constraints for trees and shrubs native to Greenland

**Figure 2.**
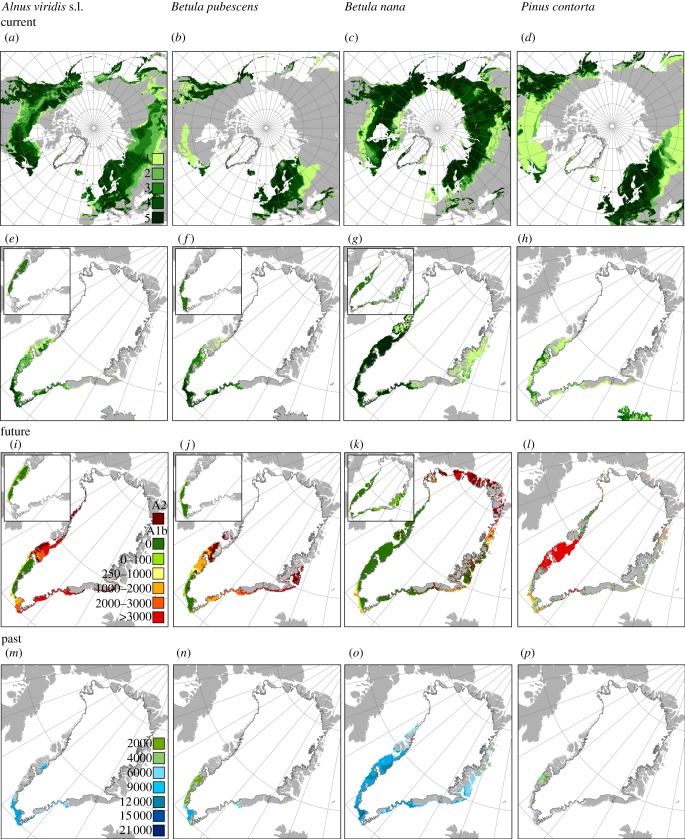
Current, future and past climatically suitable areas of three native tree and shrub species and one non-native tree species. (*a*–*d*) Projected currently suitable conditions across the Northern Hemisphere. The numbers of models (0–5) projecting presence are shown. (*e*–*h*) Projected currently suitable areas in Greenland. Insets show these areas for the regions on Greenland currently occupied by each native species (computed with a convex hull encircling all occurrence records). (*i*–*l*) Future suitable conditions (year 2100) according to the A1b and A2 scenarios and the CGCM 3.1 global circulation model. Within the areas suitable according to the A1b scenario, the time required to colonize all climatically suitable areas (i.e. migration lags in years computed based on observed migration rates and a simple distance-based approach) are shown. Likely colonized areas by the year 2100 are shown in light green. Dark green areas (year 0) represent the approximated current range. The insets in (*i*–*k*) indicate the likely colonized area by year 2100 according to the MigClim migration simulation (see the electronic supplementary material, appendix S5). (*m*–*p*) Past suitable conditions. The timing (kyr ago) of the first appearance of climatically suitable conditions is shown.

**Figure 3.**
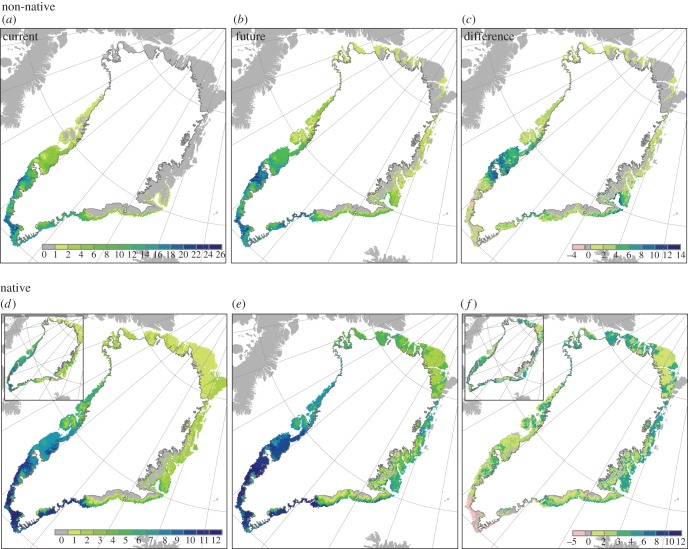
Current (*a*,*d*) and future (*b*,*e*) potential diversity, and (*c*,*f*) their difference (future minus current) for studied non-native tree and shrub species that find suitable climates in Greenland today (*a*–*c*; *n* = 26) and for all native Greenlandic tree and shrub species (*d*–*f* ; *n* = 12). Future projections are according to the A1b scenario for 2091–2100 and the CGCM 3.1 global circulation model. Insets indicate the current observed diversity (*d*) and its difference (*f*) with the future potential diversity.

**Figure 4.**
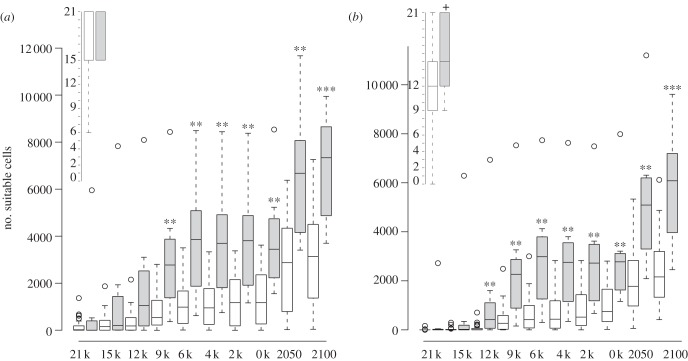
Box and whisker plots showing the change in suitable climates through time (k, kyr ago) for native Greenlandic (grey bars) and for non-native (white bars) tree and shrub species that find suitable climates in Greenland today. The number of suitable cells (10′ resolution) per species supported by at least one (*a*) or three models (*b*). The inserted box plots (top-right) indicate the timing of the appearance of the first suitable cells in Greenland for non-native and Greenlandic species. Significance tested with Wilcoxon rank sum tests: ***p < 0.001, **p < 0.01, *p < 0.05, + marginally significant (*p* = 0.0512).

When comparing the observed distribution of Greenland's native species with projected areas of suitable conditions, we find high agreement for half of the native Greenlandic species, i.e. *B. nana, E. nigrum* s.l., *J. communis*, *Rhododendron lapponicum* s.l., *Sa. glauca* and *V. uliginosum* (figure 2; electronic supplementary material, appendix S3). For the remainder, generally species with higher temperature requirements, we also project considerable areas outside the currently observed distribution as climatically suitable. These areas are, especially, in east- and southeast Greenland (*B. pubescens, So. groenlandica, Rhododendron tomentosum, A. viridis* s.l.) as well as small (*B. pubescens*) or larger (*B. glandulosa, A. viridis* s.l.; figure 2) areas around Kangerlussuaq. For *Sa. arctica*, a high Arctic species, large areas in southern Greenland, south of the species current distribution, were projected suitable. A majority of the climatic niche models (three or more) indicate that suitable climates have been present in Greenland since 9 kyr ago for *B. pubescens* and *J. communis*, since 12 kyr ago for *A. viridis* ssp. *crispa and B. glandulosa*, and since 15 kyr ago or 21 kyr ago for the rest of the native species (electronic supplementary material, appendix S3; figure 4). Given pollen-based arrival dates, immigration lags ranging between 900 and 13 000 years are estimated for the native species (see the electronic supplementary material, appendix S5). By year 2100, many species are projected to find substantial suitable areas north of their current ranges (figure 3; electronic supplementary material, appendix S3). New suitable areas and the largest increases in potential species richness are projected for central-west Greenland around Nuuk and Kangerlussuaq and northwards, as well as on the east coast, notably on Jameson Land (figure 3), also for the relatively tall-growing *B. pubescens* and *A. viridis* ssp. *crispa* (figure 2; electronic supplementary material, appendix S3). The key shrub species *B. nana* is projected to find suitable climatic conditions in most parts of ice-free Greenland by the end of this century (figure 2; electronic supplementary material, appendix S3). Nonetheless, the migration simulations and estimated likely colonized areas suggest that mainly local to regional geographical expansions are to be expected by year 2100, despite the large increases in climatically suitable areas (figures 2 and 3; electronic supplementary material, appendix S5). Furthermore, we estimate that Greenland's native species require more than 2000 years (median, *ca* 5700 years) to reach all areas climatically suitable by the year 2100 (electronic supplementary material, appendix S5) or, if these areas should be colonized by the year 2100, a migration rate of 3–29 km yr^–1^ would be required (figure 2; electronic supplementary material, appendix S5).

### (c) Climate potential and migration constraints for North American and Eurasian trees and shrubs in Greenland

The majority of the climatic niche models (three or more) support the availability of suitable climate conditions in Greenland for 46.5 per cent of the analysed North American and Eurasian species (see the electronic supplementary material, appendix S3), while 71.4 per cent was supported by at least one model. In several cases, only the minority of models project the presence of species already planted and reported to grow well in Greenland, e.g. *Betula pendula, Larix sibirica, Larix laricina, Pic. mariana*, *Picea glauca*, *Picea abies*, *Pinus sylvestris* and *Populus balsamifera* (see the electronic supplementary material, appendices S2 and S3). Generally, the models project that most of the non-native species would find suitable conditions in southern Greenland and further north along the west coast around Nuuk (figure 3). A bit further north around Kangerlussuaq, we project suitable conditions for fewer species, e.g. *Pinus contorta* (figures 2 and 3; electronic supplementary material, appendix S3). According to the majority of the climatic niche models, suitable climates became available in Greenland before 9 kyr ago for the majority (96%) of the non-native species finding suitable climates in Greenland today, suggesting immigration lags of at least 9000 years (see the electronic supplementary material, appendix S5). Climate tends to become suitable later for non-native than native species, and from 12 kya ago onwards suitable area for native species has been significantly larger than for non-native species (figure 4). Substantial additional areas, notably around Nuuk, Kangerlussuaq and Jameson Land, are projected to become suitable for many more tree and shrub species in the future (figure 3), including species of genera such as *Larix* and *Picea* that were common in Greenland in earlier warm periods (see the electronic supplementary material, appendix S3). The future estimated likely colonized areas, migration lags (minimum, *ca* 2500; median, *ca* 8500 years) and required migration rates (7–29 km yr^–1^), however, indicate that natural expansions by the year 2100 are likely to be at a local scale (figure 2; electronic supplementary material, appendix S5).

### (d) Source and sink areas with analogous climates

**Figure 5.**
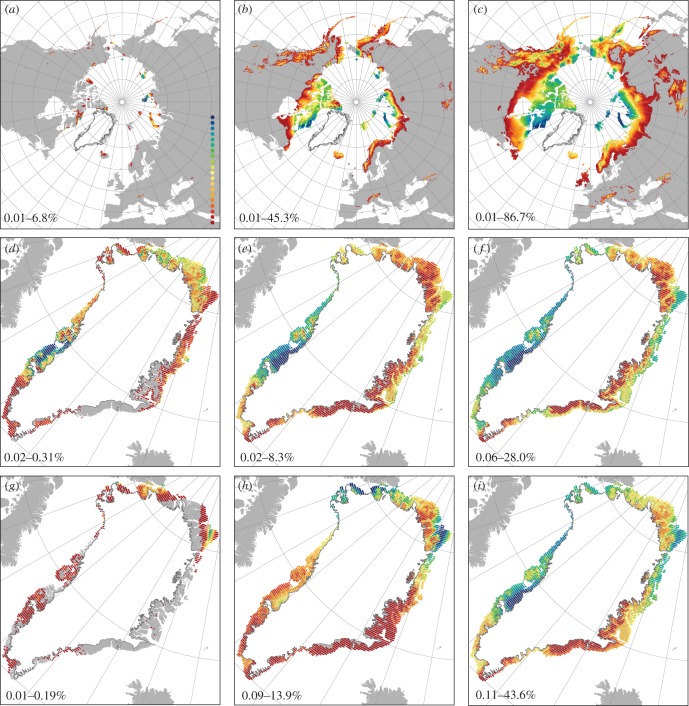
Areas of analogous climate. Analogous climates were calculated with niche breadths of 5, 25 and 50% (columns 1–3, see text and electronic supplementary material, appendix S4 for details). (*a*–*c*) Climates in North America and Eurasia analogous to those of Greenland. The value of each cell in North America and Eurasia represents the area in Greenland with analogous climate conditions, calculated as a percentage of Greenland's total ice-free area. (*d*–*f*) Climates in Greenland analogous to those of Eurasia. The value of each cell in Greenland represents the area in Eurasia with analogous climate conditions. (*g*–*i*) Climates in Greenland analogous to those of North America. The legend is plotted with Natural Breaks (Jenks). The values at the bottom of each plot give its range of values, with minimum values in red and maximum values in blue.

Potential source areas for immigrants into Greenland are areas in neighbouring regions with climates similar to those on Greenland. Eurasia has larger potential source areas than North America, but these areas are on average more distant to Greenland than those in North America (see the electronic supplementary material, appendix S4, figure S4.2). As expected, potential source areas mainly occur in the Arctic, but there are also some in mountain ranges in western North American and Scandinavia as well as in the Alps (figure 5*a*–*c*). Among potential source areas, Baffin Island, Svalbard and parts of Ellesmere Island harbour the climate conditions that are the most common in Greenland (figure 5*a*–*c*). In contrast, potential source areas in Iceland have climates that are relatively rare in Greenland. Potential sink areas for immigrants into Greenland are areas in Greenland with climates similar to those in neighbouring regions. Sink areas for North American and Eurasian species are in particular found around Kangerlussuaq and in northern Greenland. Climate conditions here are analogous to those across large parts of North America (wide niche breadths only) and Eurasia (figure 5). In contrast, eastern Greenland climates are rare across the Northern Hemisphere (figure 5).

## 4. Discussion

Greenland could be greener today. Our treeline and niche models provide evidence that suitable climatic conditions occur in Greenland for the majority of the non-native boreal species we studied. Areas climatically suitable for tree growth and several non-native species are particularly projected for southern and central-western Greenland (figures 1–3). Overall, these areas coincide with occurrences of native trees and tall shrub species, as well as successful plantings of several non-native coniferous genera (*Abies*, *Larix*, *Picea*, *Pinus*, *Thuja* and *Tsuga*) and broad-leaved trees (*Populus, Sorbus* and *Alnus*; figure 1; electronic supplementary material, appendix S2). In addition, in southern Greenland cones or fruits have been observed for 18 planted non-native tree species, resulting in self-sown offspring for at least *L. sibirica*, *Pic. glauca*, *Pinus contorta*, *Salix alaxensis* and *Po. balsamifera* (figure 1; electronic supplementary material, appendix S2). Biogeochemistry vegetation modelling [51] also supports a natural vegetation of cold-tolerant evergreen needle-leaf forest across large areas in southern Greenland. In central-western Greenland some native and non-native species even find suitable conditions in areas that naturally harbour no tree species at all. Around Kangerlussuaq, several non-native species are reported to grow relatively well (e.g. *L. sibirica, Pinus contorta, Pic. glauca, Pic. mariana, Po. balsamifera* and *Sa. alaxensis*, figure 1; electronic supplementary material, appendix S2). Additionally, the native species *B. pubescens* and *A. viridis* do not grow here naturally, although niche models, the vegetation modelling of Kaplan *et al.* [51], and plantings indicate suitable climatic conditions.

Our modelling results, together with the additional evidence discussed earlier, provide evidence for climate-range disequilibrium and strong migration lags for some native species within Greenland, as well as immigration lags for many currently non-native coniferous and broad-leaved cold-tolerant species. Among these are several species and genera that represent taxa also found in Greenland during Pliocene and certain Pleistocene warm periods, notably *Alnus*, *Betula*, *Abies*, *Picea*, *Larix*, *Myrica*, *Populus*, *Tsuga* and *Thuja* [11,26,28,29]. For example, *Pic. mariana* and the now extinct Greenlandic species *Larix groenlandii* [52] (closely related to the present-day northern larches *L. gmelinii*, *L. laricina*, *L. sibirica* *and* *L. decidua*) were important components of the rich subarctic forest–tundra that occurred in northeastern Greenland *ca* 2 million years ago. *Thuja* was also part of this vegetation. It is the western North American species *Thuja plicata* rather than the eastern North American *T. occidentalis* for which suitable areas are projected, although it was the latter that occurred in Greenland in Plio-Plioecene. Generally, among the non-native species, it is mainly species from the western North America that are projected to find large suitable areas in Greenland, e.g. *Picea sitchensis, Abies lasiocarpa, Picea engelmannii* and *Pin. contorta.* In contrast, the projected suitable areas for species with continental or eastern ranges in North America tend to be relatively small*.* This probably reflects the relative oceanic conditions in Greenland. The analyses of analogous climates support this pattern, highlighting that such climates are particularly widespread in western North America (figure 5). In summary, given its present climate southern Greenland could be greener today, with a rich boreal woody flora similar to that occurring during past warm periods (figure 3).

The expected near-future climatic changes will increase the climatic scope for the expansion of trees and shrubs in Greenland. Treeline and niche models project substantial increases in the areas climatically suitable for trees and shrubs by year 2100—expanding even to northern Greenland. Large increases in the number of native and non-native species for which there will be suitable conditions are in particular projected for central-western Greenland (around Kangerlussuaq and Nuuk) and Greenland's central-east coast (figure 3).

While climatically possible, how likely is a recolonization of Greenland by a richer boreal flora in the near future? For the tree and shrub species currently not growing in Greenland, the main question is whether they naturally can reach Greenland at all. Our findings suggest that Greenland's current tree and shrub flora is strongly shaped by postglacial immigration lags of many thousands of years. By comparing pollen-based estimates of first arrival with the time at which climate first became suitable, we estimate immigration lags of 900–13 000 years for Greenland's native tree and shrub species (see the electronic supplementary material, appendix S5). Non-native species have, by definition, failed to colonize Greenland, representing immigration lags for most species of at least 9000 years, even for those present in nearby northeast North America (electronic supplementary material, appendix S5). Generally, non-native species tend to have had less time for colonization (i.e. climate became suitable later) and the area of suitable climate available through time has been smaller than for native species (figure 4), potentially contributing to their failure to establish by natural means in Greenland. Broad geographical patterns of analogous climates in the regions around Greenland may also contribute to these immigration failures. Areas in northern Greenland and around Kangerlussuaq harbour the climatic conditions that are most widespread in North America and Eurasia, while eastern Greenland climates seem to be rare outside Greenland (figure 5). This could partly explain this region's low observed species richness [17]. Furthermore, Iceland harbours climatic conditions that are relatively rare in Greenland, especially with regard to temperature of the coldest month. Thus, Iceland and eastern Greenland seem to be poor stepping-stones for the many Eurasian species, which potentially could grow in western Greenland.

Since species currently not found in Greenland have not been able to colonize during the past several millennia, despite the presence of suitable climatic conditions in Greenland, it is unlikely that these species will do so in the near future. The Last Interglacial offers an interesting analogy here. During this period, temperatures in Greenland were 4–6°C warmer than today and within the range of what is expected for Greenland in year 2100 [2]. The climate was so mild that the temperate fern *Osmunda* was common in southern Greenland [28]. Still, the vegetation was dominated by *Alnus*, and perhaps *Betula* (cf. [30]), while conifers appear to have been absent throughout this approx. 10 000 year period [28], despite the existence of a climate that must have been suitable for many conifer species. Hence, migration constraints may strongly shape vegetation development on even multi-millennial time scales.

For native and non-native tree and shrub species already growing in Greenland, it is equally unlikely that the projected expansions will rapidly occur naturally on a Greenland-wide scale during the current century. Our migration modelling suggests future migration lags of several millennia and locally restricted spread by year 2100 (figures 2 and 3). Hence, the near-future vegetation development is likely constrained considerably by lags in dispersal, as well as other processes, such as soil development and succession, together leading to long-term disequilibrium and spatially variable vegetation dynamics [21]. Long, geographically variable delays in treeline expansion are also suggested by simulated treeline expansion lags of 150–250 years in Alaska [25] and millennia in Siberia [53], and by palaeoecological estimates of multi-millennial lags in expansions of certain tree species at some treelines in northern Canada [14,23,24]. Importantly, colonization of *B. pubescens* onto glacier forelands in Norway today takes up to 200 years below the treeline, but was delayed by a further 450 years after the initial Holocene warming, perhaps reflecting long distances to source populations [54]. Even when climate is suitable and seed sources present within local landscapes, succession to forest on deglaciated terrain often takes many decades or centuries [54,55].

Many factors might reduce and/or enhance future migration rates; topographic barriers [13,14], fragmentation [56], microsite availability, physical disturbance regimes (e.g. fire [57,58]), permafrost degradation [59], human disturbance [60] and biotic interactions (e.g. herbivory, animal burrowing, seed dispersers and competition). Notably, herbivory has recently been highlighted as an important process limiting shrub expansion in Greenland [61]. At the same time, populations of large avian and mammalian herbivores, permafrost, runoff, fire and many other factors create disturbances potentially enhancing seedling establishment [62,63]. In fact, seedling establishment is regarded as a key constraint on reproduction in Arctic plant species [64] and could be a major determinant of the speed and extent of future expansion of shrubs and trees in the Arctic. The importance of competition and fragmentation has been highlighted by modelling studies taking population dynamics, competition and dispersal into account [56,65], and several studies indicate that topographic barriers may strongly delay treeline advances [13,14]. The potential migration paths along the rugged coastline of Greenland are highly fragmented, and the future potential habitats of many species are isolated. In consequence, the species will not easily reach these fragmented habitats by natural migration, similar to the strong dispersal limitation modelled for alpine plants with isolated occurrences in the Alps [66].

Intentional plantings and unintentional anthropogenic seed transport are likely to speed up migration rates of some species, and increased economic and societal interests in remote areas of Greenland (e.g. for exploration of mineral extraction, tourism and research) will further increase the likelihood of invasions [67]. Our identification of vast areas with Greenland-analogous climate conditions across the Northern Hemisphere highlight potential source areas for future introductions into Greenland. For example, the mid-latitude mountainous areas in western North America are climatically more connected to Greenland than those of Eurasia (figure 5). Areas around the international airport in Kangerlussuaq are potentially important regions for species introduction as they have climatic conditions that are widespread across large areas in North America and Eurasia (figure 5). Our results underline that establishment of unintentionally introduced seeds may become more likely in the near future as more non-native species will find increasingly large suitable areas in Greenland and may have strong impacts on the realized vegetation changes by speeding up migration rates. Nevertheless, a more predictable source of spread is the already planted and naturalizing tree and shrub species, which could produce fast local expansions in certain areas, e.g. landscapes around urban areas. With a warmer climate, plantings of trees and shrubs as ornamentals and for forestry are likely to become more widespread in Greenland, and thus reduce future migration lags [68].

The analyses presented here are not without uncertainties. While some of the climate niche models failed to project the distribution of species already growing in Greenland, other models probably projected areas of suitable conditions that were too broad, since foehn wind systems, soils and extreme events were not considered. Hence, some species might actually only occur within sheltered valleys within the areas projected as suitable. It has been argued that important climatic factors limiting tree survival and growth in Greenland include the frequently occurring, desiccating foehn wind events and sequences of unusual cold summers rather than the cool average temperatures [18]. This idea is supported by severe injuries on both native *Betula* and planted conifers following cool summers in 1983–1984 [19]. The degree of damage due to dry foehn wind from the inland icecap can vary substantially with local topography and might necessitate finer spatial and temporal scale climate data than used here [69]. Other limitations for past and future species projections consist of the extent of the Greenland ice sheet through time as all projections are made under present-day ice extent. Ice extent in the past was obviously bigger [31], while the ice sheet is expected to retreat in the future [70]. Further limitations originate from uncertainties related with past climate simulations. It is also important to note that the geographical seed origin might play an important role for the survival of planted trees [18]. Climate niche models for a species as a whole might yield distinct projections from models of genetically distinct populations [71,72]. Such variation between populations was not taken into account here.

Several areas with known tree growth in southern Greenland and around Nuuk are not predicted by the treeline model, indicating that the model provides a conservative estimate of the potential for tree growth across Greenland. There may be several explanations for these underpredictions. First, the algorithm predicts core areas for tree growth (i.e. where population can persist over long periods due to stable climate conditions over time); hence, single individuals and smaller stands of trees may be expected beyond the limits predicted by the model, and occurrences of *B. pubescens* and *So. groenlandica* are clearly found outside the predicted areas (figure 1). Secondly, the model might fail to identified small areas with suitable microtopograhic conditions in regions with large elevational differences [68] due to larger uncertainty in the macro-scale climate data and the likely violation of the assumption of a constant adiabatic temperature gradient in topographically heterogeneous regions. This probably explains the failure of the treeline model to predict the lush native forest–shrub vegetation of the Qinngua valley in southern Greenland, as it is surrounded by high mountain peaks in a topographically heterogeneous region. Despite these uncertainties, we consider our conclusions robust due to the broadly consistent results from the treeline algorithm and the broad range of climatic niche modelling algorithms, as well as their general consistency with areas of native woodland or planted trees.

In conclusion, future warming is likely to allow growth of trees and shrubs across much of ice-free Greenland by year 2100, and provide the potential for local expansion of subarctic shrub ecosystems and boreal forest ecosystems in many parts of the southern half of the region. Such expansion would strongly affect Arctic biodiversity and ecosystems, with feedbacks to the global climate system [8]. Shade-intolerant species may be particularly vulnerable. Loss of herbaceous species has already been documented in response to increasing shrub cover [73–76] and richness of vascular plants, mosses, as well as lichens tends to decrease from medium-productive tundra to highly productive shrub-rich tundra [77]. At the same time, the three-dimensional structure of expanding shrub vegetation might favour richer and potentially more specialized animal and vertebrate communities [78–80]. Full realization of the projected expansions is likely to take centuries or even millennia in many parts of Greenland. Immigration lags, within-region dispersal limitation and related disequilibrium dynamics, including the often multi-decadal or -centennial times needed for succession into tundra or deglaciated areas—even after local species arrival—will lead to long protracted disequilibrium dynamics in Greenland's future vegetation. Thus, vegetation dynamics will be highly variable in space, contingent on accessibility to natural colonization, stochastic long-distance dispersal events and with human introductions probably playing a major role.

## References

[1] Callaghan TV 2005 Arctic tundra and polar desert ecosystems. In Arctic Climate Impact Assessment (ACIA) (eds C Symon, L Arris, B Heal), pp. 243–352 Cambridge, UK: Cambridge University Press

[2] Masson-Delmotte V 2012 Greenland climate change: from the past to the future. Wiley Interdiscip. Rev. Clim. Change 3, 427–449 (doi:10.1002/wcc.186)

[3] Tape K Sturm M Racine C 2006 The evidence for shrub expansion in Northern Alaska and the Pan-Arctic. Glob. Change Biol. 12, 686–702 (doi:10.1111/j.1365-2486.2006.01128.x)

[4] Sturm M Racine C Tape K Cronin TW Caldwell RL Marshall J 2001 Increasing shrub abundance in the Arctic. Nature 411, 546–547 (doi:10.1038/35079180) 11385559 10.1038/35079180

[5] Beck PSA Juday GP Alix C Barber VA Winslow SE Sousa EE Heiser P Herriges JD Goetz SJ 2011 Changes in forest productivity across Alaska consistent with biome shift. Ecol. Lett. 14, 373–379 (doi:10.1111/j.1461-0248.2011.01598.x) 21332901 10.1111/j.1461-0248.2011.01598.x

[6] Suarez F Binkley D Kaye M Stottlemyer R 1999 Expansion of forest stands into tundra in the Noatak National Preserve, northwest Alaska. Ecoscience 6, 465–470

[7] Lloyd AH 2005 Ecological histories from Alaskan tree lines provide insight into future change. Ecology 86, 1687–1695

[8] Chapin FS III 2005 Role of land-surface changes in Arctic summer warming. Science 310, 657–660 (doi:10.1126/science.1117368) 16179434 10.1126/science.1117368

[9] Post E 2009 Ecological dynamics across the Arctic associated with recent climate change. Science 325, 1355–1358 (doi:10.1126/science.1173113) 19745143 10.1126/science.1173113

[10] Miller GH 2010 Temperature and precipitation history of the Arctic. Q. Sci. Rev. 29, 1679–1715 (doi:10.1016/j.quascirev.2010.03.001)

[11] Bennike O Böcher J 1990 Forest-tundra neighbouring the North Pole: plant and insect remains from the Plio-Pleistocene Kap København formation, North Greenland. Arctic 43, 331–338

[12] Elmendorf SC 2012 Plot-scale evidence of tundra vegetation change and links to recent summer warming. Nat. Clim. Change 2, 453–457 (doi:10.1038/nclimate1465)

[13] Rupp TS Chapin FS III Starfield AM 2001 Modeling the influence of topographic barriers on treeline advance at the forest-tundra ecotone in northwestern Alaska. Clim. Change 48, 399–416 (doi:10.1023/a:1010738502596)

[14] Payette S 2007 Contrasted dynamics of northern Labrador tree lines caused by climate change and migrational lag. Ecology 88, 770–780 (doi:10.1890/06-0265) 17503604 10.1890/06-0265

[15] Fredskild B Ødum S 1990 The Greenland mountain birch zone, an introduction. Bioscience 33, 3–7

[16] Walker DA 2005 The Circumpolar Arctic vegetation map. J. Veg. Sci. 16, 267–282 (doi:10.1111/j.1654-1103.2005.tb02365.x)

[17] Elven R Murray DF Rabizzhivin VY Yurtsev BA 2011 Annotated checklist of the Panarctic flora (PAF) vascular plants. See http://nhm2.uio.no/paf/

[18] Ødum S 1991 Choice of species and origins for arboriculture in Greenland and the Faroe Islands. Dansk Dendrologisk Forening 9, 3–78

[19] Ødum S 1990 Afforestation experiments reflecting the treeline conditions in Southwest Greenland. Bioscience 33, 43–61

[20] Normand S Ricklefs RE Skov F Bladt J Tackenberg O Svenning J-C 2011 Postglacial migration supplements climate in determining plant species ranges in Europe. Proc. R. Soc. B 278, 3644–3653 (doi:10.1098/rspb.2010.2769) 10.1098/rspb.2010.2769 PMC3203492 21543356

[21] Svenning J-C Sandel B In press. Disequilibrium vegetation dynamics under future climate change. Am. J. Bot. (doi:10.3732/ajb.1200469) 10.3732/ajb.1200469 23757445

[22] Neilson RP Pitelka LF Solomon AM Nathan R Midgley GF Fragoso JMV Lischke H Thompson K 2005 Forecasting regional to global plant migration in response to climate change. BioScience 55, 749 (doi:10.1641/0006-3568(2005)055[0749:FRTGPM]2.0.CO;2)

[23] Laliberté A-C Payette S 2008 Primary succession of subarctic vegetation and soil on the fast-rising coast of eastern Hudson Bay, Canada. J. Biogeogr. 35, 1989–1999 (doi:10.1111/j.1365-2699.2008.01932.x)

[24] Caccianiga M Payette S 2006 Recent advance of white spruce (*Picea glauca*) in the coastal tundra of the eastern shore of Hudson Bay (Québec, Canada). J. Biogeogr. 33, 2120–2135 (doi:10.1111/j.1365-2699.2006.01563.x)

[25] Johnstone J Chapin F 2003 Non-equilibrium succession dynamics indicate continued northern migration of lodgepole pine. Glob. Change Biol. 9, 1401–1409 (doi:10.1046/j.1365-2486.2003.00661.x)

[26] Funder S Abrahamsen N Bennike O Feyling-hanssen RW 1985 Forested Arctic: evidence from North Greenland. Geology 13, 542–546 (doi:10.1130/0091-7613(1985)13<542)

[27] Bennike O Knudsen KL Abrahamsen N Böcher J Cremer H Wagner B 2010 Early Pleistocene sediments on store Koldewey, Northeast Greenland. Boreas 39, 603–619 (doi:10.1111/j.1502-3885.2010.00147.x)

[28] De Vernal A Hillaire-Marcel C 2008 Natural variability of Greenland climate, vegetation, and ice volume during the past million years. Science 320, 1622–1625 (doi:10.1126/science.1153929) 18566284 10.1126/science.1153929

[29] Willerslev E 2007 Ancient biomolecules from deep ice cores reveal a forested southern Greenland. Science 317, 111–114 (doi:10.1126/science.1141758) 17615355 10.1126/science.1141758 PMC2694912

[30] Bennike O Böcher J 1994 Land biotas of the last interglacial/glacial cycle on Jameson Land, East Greenland. Boreas 23, 479–487 (doi:10.1111/j.1502-3885.1994.tb00615.x)

[31] Böcher J 2012 Interglacial insects and their possible survival in Greenland during the last glacial stage. Boreas 41, 644–659 (doi:10.1111/j.1502-3885.2012.00251.x)

[32] Westergaard KB Alsos IG Popp M Engelskjø T Flatberg KI Brochmann C 2011 Glacial survival may matter after all: Nunatak signatures in the rare European populations of two west-Arctic species. Mol. Ecol. 20, 376–393 (doi:10.1111/j.1365-294X.2010.04928.x) 21156004 10.1111/j.1365-294X.2010.04928.x

[33] Hoffmann MH 2011 Not across the North Pole: plant migration in the Arctic. New Phytol. 193, 474–480 (doi:10.1111/j.1469-8137.2011.03924.x) 21988606 10.1111/j.1469-8137.2011.03924.x

[34] Bennike O 1999 Colonisation of Greenland by plants and animals after the last ice age: a review. Polar Rec. 35, 323–336 (doi:10.1017/S0032247400015679)

[35] Fredskild B 1991 The genus *Betula* in Greenland: Holocene history, present distribution and synecology. Nordic J. Bot. 11, 393–412 (doi:10.1111/j.1756-1051.1991.tb01236.x)

[36] Chapman AD 2005 Principles of data quality. version 1.0. Report for the Global Biodiversity Information Facility. GBIF, Copenhagen, Denmark

[37] Singarayer JS Valdes PJ 2010 High-latitude climate sensitivity to ice-sheet forcing over the last 120kyr. Q. Sci. Rev. 29, 43–55 (doi:10.1016/j.quascirev.2009.10.011)

[38] Maiorano L 2012 Building the niche through time: using 13,000 years of data to predict the effects of climate change on three tree species in Europe. *Glob. Ecol. Biogeogr.*, 22, 302–317 (doi:10.1111/j.1466-8238.2012.00767.x)

[39] Randall D 2007 Climate models and their evaluation. In Climate Change 2007: The Physical Science Basis. Contribution of Working Group I to the Fourth Assessment Report of the Intergovernmental Panel on Climate Change (eds Solomon S Qin D Manning M Chen Z Marquis M Averyt KB Tignor M Miller HL), ch. 8, pp. 589–662 Cambridge, UK: Cambridge University Press

[40] Engler R Hordijk W Guisan A 2012 The MIGCLIM R package-seamless integration of dispersal constraints into projections of species distribution models. Ecography 35, 872–878 (doi:10.1111/j.1600-0587.2012.07608.x)

[41] Körner C Paulsen J Spehn EM 2011 A definition of mountains and their bioclimatic belts for global comparisons of biodiversity data. Alpine Bot. 121, 73–78 (doi:10.1007/s00035-011-0094-4)

[42] Körner C Paulsen J 2004 A world-wide study of high altitude treeline temperatures. J. Biogeogr. 31, 713–732 (doi:10.1111/j.1365-2699.2003.01043.x)

[43] Elith J 2006 Novel methods improve prediction of species’ distributions from occurrence data. Ecography 29, 129–151 (doi:10.1111/j.2006.0906-7590.04596.x)

[44] Barbet-Massin M Jiguet F Albert CH Thuiller W 2013 Selecting pseudo-absences for species distribution models: how, where and how many? Methods Ecol. Evol. 3, 327–338 (doi:10.1111/j.2041-210X.2011.00172.x)

[45] Allouche O Tsoar A Kadmon R 2006 Assessing the accuracy of species distribution models: prevalence, kappa and the true skill statistic (TSS). J. Appl. Ecol. 43, 1223–1232 (doi:10.1111/j.1365-2664.2006.01214.x)

[46] Fielding AH Bell JF 1997 A review of methods for the assessment of prediction errors in conservation presence/absence models. Environ. Conserv. 24, 38–49 (doi:10.1017/S0376892997000088)

[47] Thuiller W Georges D Engler R 2012 Biomod2: ensemble platform for species distribution modeling See http://cran.r-project.org/web/packages/biomod2/index.html

[48] R Core Team 2012 R: a language and environment for statistical computing. Vienna, Austria: R Foundation for Statistical Computing.

[49] Phillips S Anderson R Schapire R 2006 Maximum entropy modeling of species geographic distributions. Ecol. Model. 190, 231–259 (doi:10.1016/j.ecolmodel.2005.03.026)

[50] Ohlemüller R Huntley B Normand S Svenning J-C 2012 Potential source and sink locations for climate-driven species range shifts in Europe since the Last Glacial Maximum. Glob. Ecol. Biogeogr. 21, 152–163 (doi:10.1111/j.1466-8238.2011.00674.x)

[51] Kaplan JO 2003 Climate change and Arctic ecosystems. II. Modeling, paleodata-model comparisons, and future projections. J. Geophys. Res. 108, 8171 (doi:10.1029/2002JD002559)

[52] Schorn H 1994 A preliminary discussion of fossil larches (*Larix*, Pinaceae) from the Arctic. Science 22/23, 173–183

[53] Epstein H Kaplan JO Lischke H Yu Q 2007 Simulating future changes in Arctic and subarctic vegetation. Comput. Sci. Eng. 9, 12–23 (doi:10.1109/MCSE.2007.84)

[54] Birks HJB Birks HH 2008 Biological responses to rapid climate change at the Younger Dryas–Holocene transition at Kråkenes, western Norway. Holocene 18, 19–30 (doi:10.1177/0959683607085572)

[55] Chapin FS III Walker LR Fastie C Sharman L 1994 Mechanisms of primary succession following deglaciation at Glacier Bay, Alaska. Ecol. Model. 64, 149–175

[56] Meier ES Lischke H Schmatz DR Zimmermann NE 2012 Climate, competition and connectivity affect future migration and ranges of European trees. Glob. Ecol. Biogeogr. 21, 164–178 (doi:10.1111/j.1466-8238.2011.00669.x)

[57] Kasischke ES Turetsky MR 2006 Recent changes in the fire regime across the North American boreal region—spatial and temporal patterns of burning across Canada and Alaska. Geophys. Res. Lett. 33, 1–5 (doi:10.1029/2006GL025677)

[58] Mack MC Bret-Harte MS Hollingsworth TN Jandt RR Schuur EaG Shaver GR Verbyla DL 2011 Carbon loss from an unprecedented Arctic tundra wildfire. Nature 475, 489–492 (doi:10.1038/nature10283) 21796209 10.1038/nature10283

[59] Lawrence DM Slater AG Tomas Ra Holland MM Deser C 2008 Accelerated Arctic land warming and permafrost degradation during rapid sea ice loss. Geophys. Res. Lett. 35, L11506 (doi:10.1029/2008GL033985)

[60] Kemper J Macdonald S 2009 Directional change in upland tundra plant communities 20–30 years after seismic exploration in the Canadian low-arctic. J. Veg. Sci. 20, 557–567

[61] Post E Pedersen C 2008 Opposing plant community responses to warming with and without herbivores. Proc. Natl Acad. Sci. USA 105, 12 353–12 358 (doi:10.1073/pnas.0802421105) 10.1073/pnas.0802421105 PMC2527915 18719116

[62] Forbes BC Ebersole JJ Strandberg B 2001 Anthropogenic disturbance and patch dynamics in circumpolar Arctic ecosystems. Conserv. Biol. 15, 954–969 (doi:10.1046/j.1523-1739.2001.015004954.x)

[63] Lantz TC Marsh P Kokelj SV 2012 Recent shrub proliferation in the Mackenzie Delta uplands and microclimatic implications. Ecosystems 16, 47–59 (doi:10.1007/s10021-012-9595-2)

[64] Graae BJ Ejrnæs R Lang SI Meineri E Ibarra PT Bruun HH 2011 Strong microsite control of seedling recruitment in tundra. Oecologia 166, 565–576 (doi:10.1007/s00442-010-1878-8) 21170749 10.1007/s00442-010-1878-8 PMC3094527

[65] Collingham Y Huntley B 2000 Impacts of habitat fragmentation and patch size upon migration rates. Ecol. Appl. 10, 131–144

[66] Dullinger S 2012 Extinction debt of high-mountain plants under twenty-first-century climate change. Nat. Clim. Change 2, 1–4 (doi:10.1038/nclimate1514)

[67] Chown SL 2012 Continent-wide risk assessment for the establishment of nonindigenous species in Antarctica. Proc. Natl Acad. Sci. USA 109, 1–6 (doi:10.1073/pnas.1119787109) 10.1073/pnas.1119787109 PMC3323995 22393003

[68] Van der Veken S Hermy M Vellend M Knapen A Verheyen K 2008 Garden plants get a head start on climate change. Front. Ecol. Environ. 6, 212–216 (doi:10.1890/070063)

[69] Zimmermann NE Yoccoz NG Edwards TC Meier ES Thuiller W Guisan A Schmatz DR Pearman PB 2009 Climatic extremes improve predictions of spatial patterns of tree species. Proc. Natl Acad. Sci. USA 106 (Suppl.), 19 723–19 728 (doi:10.1073/pnas.0901643106) 10.1073/pnas.0901643106 PMC2780931 19897732

[70] Alley RB 2010 History of the Greenland ice sheet: paleoclimatic insights. Q. Sci. Rev. 29, 1728–1756 (doi:10.1016/j.quascirev.2010.02.007)

[71] Pearman PB D'Amen M Graham CH Thuiller W Zimmermann NE 2010 Within-taxon niche structure: niche conservatism, divergence and predicted effects of climate change. Ecography 33, 990–1003 (doi:10.1111/j.1600-0587.2010.06443.x)

[72] D'Amen M Zimmermann NE Pearman PB 2012 Conservation of phylogeographic lineages under climate change. Glob. Ecol. Biogeogr. 22, 93–104 (doi:10.1111/j.1466-8238.2012.00774.x)

[73] Wilson SD Nilsson C 2009 Arctic alpine vegetation change over 20 years. Glob. Change Biol. 15, 1676–1684 (doi:10.1111/j.1365-2486.2009.01896.x)

[74] Pajunen AM Oksanen J Virtanen R 2011 Impact of shrub canopies on understorey vegetation in western Eurasian tundra. J. Veg. Sci. 22, 837–846 (doi:10.1111/j.1654-1103.2011.01285.x)

[75] Callaghan TV 2013 Ecosystem change and stability over multiple decades in the Swedish sub-arctic: complex processes and multiple drivers. Phil. Trans. R. Soc. B 368, 20120488 (doi:10.1098/rstb.2012.0488) 23836792 10.1098/rstb.2012.0488 PMC3720059

[76] Olofsson J te Beest M Ericson L 2013 Complex biotic interactions drive long-term vegetation dynamics in a subarctic ecosystem. Phil. Trans. R. Soc. B 368, 20120486 (doi:10.1098/rstb.2012.0486) 23836791 10.1098/rstb.2012.0486 PMC3720058

[77] Virtanen R Grytnes J-A Lenoir J Luoto M Oksanen J Oksanen L Svenning J-C 2012 Productivity–diversity patterns in Arctic tundra vegetation. Ecography 35, 331–341 (doi:10.1111/j.1600-0587.2012.07903.x)

[78] Sokolov V Ehrich D Yoccoz NG Sokolov A Lecomte N 2012 Bird communities of the Arctic shrub tundra of Yamal: habitat specialists and generalists. PLoS ONE 7, e50335 (doi:10.1371/journal.pone.0050335) 23239978 10.1371/journal.pone.0050335 PMC3519781

[79] Ims RA Henden J-A 2012 Collapse of an Arctic bird community resulting from ungulate-induced loss of erect shrubs. Biol. Conserv. 149, 2–5 (doi:10.1016/j.biocon.2012.02.008)

[80] Ehrich D 2012 The importance of willow thickets for ptarmigan and hares in shrub tundra: the more the better? Oecologia 168, 141–151 (doi:10.1007/s00442-011-2059-0) 21833646 10.1007/s00442-011-2059-0

